# Supplementary material for: MetaRibo-Seq measures translation in microbiomes
Source: Nat Commun. 2020 Jun 29;11:3268. doi: 10.1038/s41467-020-17081-z (PMC7324362; doi:10.1038/s41467-020-17081-z)
Supplement: Supplementary file 10 — Supplementary Data 7 [file 41467_2020_17081_MOESM10_ESM.zip › File2/Confidence_VeryHigh_Taxonomy/44115_out.krona.html]

Javascript must be enabled to view this page.

members
magnitude
magnitudeUnassigned
count
unassigned
taxon
rank

44115\_out

5

1

SRS075341\_contig\_number\_contig-100\_2925.127345

superkingdom
4
2

phylum
1
544448

31969
1
class

1
order
186329


SRS058070\_contig\_number\_30521
2053494
1
species

phylum
3
1239

186801
3
class

186802
3
order

family
3
186806

1730
genus
3

142586

SRS017821\_contig\_number\_23132SRS058070\_contig\_number\_30159SRS075341\_contig\_number\_10604
species
3
